# The promise and the reality: a mental health workforce perspective on technology-enhanced youth mental health service delivery

**DOI:** 10.1186/s12913-016-1790-y

**Published:** 2016-10-10

**Authors:** Simone Orlowski, Sharon Lawn, Ben Matthews, Anthony Venning, Kaisha Wyld, Gabrielle Jones, Megan Winsall, Gaston Antezana, Geoffrey Schrader, Niranjan Bidargaddi

**Affiliations:** 1 Flinders Human Behaviour & Health Research Unit, Flinders University, Margaret Tobin Centre, FMC, Sturt Road, Bedford Park, Adelaide, SA 5042 Australia; 2 Young and Well, Cooperative Research Centre, Abbotsford, VIC Australia; 3 School of Information Technology & Electrical Engineering, University of Queensland, St Lucia, Australia; 4 Country and Outback Health, Kadina, Australia; 5 Personal Health Informatics, School of Medicine, Flinders University, Adelaide, Australia

**Keywords:** Rural Youth, Mental Health, Technology, Implementation, Design

## Abstract

**Background:**

Digital technologies show promise for reversing poor engagement of youth (16–24 years) with mental health services. In particular, mobile and internet based applications with communication capabilities can augment face-to-face mental health service provision. The literature in this field, however, fails to adequately capture the perspectives of the youth mental health workforce regarding utility and acceptability of technology for this purpose.

**Methods:**

This paper describes results of in-depth qualitative data drawn from various stakeholders involved in provision of youth mental health services in one Australian rural region. Data were obtained using focus groups and semi-structured interviews with regional youth mental health clinicians, youth workers and support/management staff (*n* = 4 focus groups; *n* = 8 interviews) and analysed via inductive thematic analysis.

**Results:**

Results question the acceptability of technology to engage clients within youth mental health services. Six main themes were identified: *young people in a digital age, personal connection, power and vulnerability, professional identity, individual factors* and *organisational legitimacy*.

**Conclusions:**

These findings deepen the understanding of risks and challenges faced when adopting new technologies in mental healthcare. Recommendations for technology design and implementation in mental health services are made.

**Electronic supplementary material:**

The online version of this article (doi:10.1186/s12913-016-1790-y) contains supplementary material, which is available to authorized users.

## Background

Mental health-related problems account for a significant proportion of the disease burden in young Australians [1]. It is reported that over 20 % of young Australians (15–19 years) meet the criteria for having a probable mental illness, and 60 % of these report to be *uncomfortable* in seeking help or advice for mental illness [2]. Accordingly, it could be said that the individuals who would most benefit from formal mental health assistance do not access it. This failure to engage with mental health services has been attributed to many well-established barriers which include stigma and negative attitudes toward help-seeking, a preference for self-reliance and/or informal sources of help (e.g. friends and family), along with limited mental health literacy and emotional competence [3, 4]. Additionally, geographic and financial barriers can further amplify difficulties associated with help-seeking (e.g., adolescents in small rural communities with limited financial flexibility and availability of transport). With this in mind, a growing body of literature champions the introduction of more affordable, accessible and acceptable health services and support for Australians via technology-related solutions [5–9].

Technology solutions widely studied for mental health provision mostly include internet based self-help programs, like internet based Cognitive Behavioural Therapy interventions (iCBT’s) for treating mild to moderate mental health problems (e.g. depression and anxiety) or mobile apps for self-management and treatment which require limited or no interaction with health professionals. The evidence for iCBTs to address mild to moderate mental disorders is compelling [10], and, as such, they are now recognised by national review bodies e.g. National Registry of Evidence-Based Programs and Practices in the USA and NICE UK) [11]. Telepscyhiatry (i.e. mental health service provision via video-conference) is also well established [12], an approach particularly well suited to the more severe spectrum of disorders which require specialist professional input.

In contrast, relatively little is known about how and which of various technologies can augment traditional face-to-face mental health services, particularly to improve young people’s engagement with and navigation of the broader mental health system [11, 13]. For example, mobile and internet based communication permeate lives of young adults [14] but traditional mental health service provision is face-to-face based. The limited number of prior studies situated in a mental health context suggest factors such as a lack of organisational buy-in and readiness have negatively affected uptake of technology (e.g., no strategic planning, limited leadership and inappropriate funding) [15–18]. Moreover, human factors such as clinician concerns around lack of clinical utility, suitability of consumers, technical skills and links to current workflow and practices were reported as barriers to routine uptake in practice [16, 18]. Similar results have been reported in pre-implementation studies in youth mental health service contexts [13, 19]. This body of literature suggests an unbalanced focus on the technical components of design over human and organisational factors. Evidence from the telehealth literature has demonstrated that clinician acceptance, along with workforce demand and availability, adequate technology resourcing and project champions are key factors in establishment of sustainable telehealth services [20, 21].

Despite some willingness to incorporate technology in mental health practice, its use with consumers is not widespread [13, 19] and arguably underused by specialised mental health professionals [22, 23]. Moreover, technology adoption in professional mental health settings has not kept pace with the rate of non-professional use, presumably because clinicians have reported a lack of awareness of the options available [24, 25]. Taken together, this evidence suggests that use of technology to engage consumers is, by and large, considered outside standard practice by youth mental health service providers [13, 19]. In fact, low rates of technology use has been reported in CBT therapists, youth workers and rural health practitioners [11]. Recent research with rural healthcare providers (which included psychologists, psychiatrists, clinical social workers and general practitioners) has suggested an adjunct role for technology in service provision. Factors such as ease of use, time required, access to appropriate professional development and impact on therapeutic relationships have been reported as factors affecting uptake of technology-based tools [26].

With the above in mind, a more nuanced appreciation of mental health work culture is required. For example, individuals currently providing mental health services are primarily tasked with ongoing risk identification, assessment, management and reduction of mental illness symptoms, with allocation of resources and training reflective of the significant priority placed on these endeavours [27, 28]. The structure and nature of the mental health system is primarily shaped “by risk and the imperative to manage it” [29]. While some innovative examples of use of technology to assist practice exist [30–34], the reported clinician preference for face-to-face service provision over technology-based interaction may be reflective, in part, of the risk-focussed culture in which they work [13, 19].

An increasingly technology-focussed style of mental health service provision is also at odds with the traditional power distribution and hierarchy in healthcare [35], and more specifically mental healthcare, which has a history of positioning consumers as disempowered participants [36–40]. In contrast to this, a technology-based style of engagement implies a shift in power away from the clinician toward the consumer, and a focus on their needs and preferences. A further complicating factor with respect to technology implementation with face-to-face mental health services is the heterogeneous nature of youth mental health service provision in Australia. The different organisations that deliver youth mental health services generally work within distinctly different models of practice. In turn, these differences then impact on the individual clinician’s mindset. For example, interactions with young people can be primarily therapeutic or alternatively case management in nature. Additionally, some organisations run a dedicated youth service, whereas others deliver a serve spanning multiple age groups. Services can also differ with respect to whether they implement an illness or strengths based service model, and whether their primary focus is clinical or psychosocial in nature.

### The current study

Previous research around the role of technology in face-to-face youth mental health services has been quantitative in nature, driven by selected samples at an organisational level [19], or involving a convenience sample of clinicians who were likely early adopters of technology in clinical practice [13]. Furthermore, research with rural mental healthcare providers has focused on the individual perspectives of private care providers [26]. Thus, the literature in this field fails to adequately capture perspectives of the broader youth mental health workforce regarding their receptiveness and readiness toward use of mobile and online-based technologies in their work with young people, particularly to improve their engagement with and navigation of the broader mental health system. The current study, therefore, aimed to contribute to the literature via an in-depth, holistic and systemic research approach. To achieve this, it sought the perspectives of existing government-based frontline teams servicing different tiers of the youth mental health system. Their perspectives were balanced by the views of individuals working in middle and upper level management roles and in more general youth-based services.

## Methods

### Study Setting

Technology used by clinicians when working with consumers is influenced by the type of mental health services available as well as integration between them [41]. The Australian mental health system is comprised of three tiers. Tier one: Primary care - often the first point of reference for help seeking consisting of those individuals/services and informal supports with no formal mental health training. Tier two: Specialist care with mental health expertise; these individuals/services tend to see clients with moderate to severe mental health disorders or those at risk of developing one. Tier three: Specialist mental health services; these services are multidisciplinary in nature; they provide varying levels crisis response and assertive outreach (and inpatient services) to clients who may present as difficult to engage and/or have complex needs [42]. Each of the tiers of the system were represented in this study.

### Participants

Data were collected from general and mental health youth workers from one rural South Australian region. More than three quarters (77 %) of the 1.65 million people living in South Australia reside in the greater Adelaide area and the remainder are widely dispersed throughout the non-metropolitan landscape measuring 982,380 km^2^ [43]. Geographical pressures are, therefore, part of providing mental health services in the state. The region sampled in the current study was chosen because the availability and distribution of its major stakeholders in youth mental services reflected that of similar services across South Australia’s other rural areas. Participants were recruited to four focus groups (*n* = 40 participants) and semi-structured interviews (*n* = 8 participants). Refer to Tables 1 and 2 for a description of focus group participants. Interview participants were comprised of three youth mental health clinicians (one female): two were social workers and one mental health nurse (two of which were aged 18–40 years and the third 40+ years); and five support and managerial/executive staff (three female): two were executive level management, one middle level management and two project officers (20 % aged 18–40 years and 80 % 40+ years). The study received clearance from the South Australian Department of Health Human Research Ethics Committee (HREC/14/SAH/34).

**Table 1:** Focus group demographic information

Number	Type	Composition	Number of participants	Gender	Age
1	mental health service 1	existing team	14	70 % female	18–29 (33 %)
					40+ (67 %)
2	mental health service 2	existing team	7	86 % female	18–39 (71 %)
					40+ (29 %)
3	mental health service 3	existing team	13	62 % female	18–39 (8 %)
					40+ (92 %)
4	Youth service workers	various	6	50 % female	18–39 (50 %)
					40+ (50 %)

**Table 2:** Profession of focus group participants

Profession	Percentage*
Social worker	17 %
Mental health nurse	19 %
Psychologist	7 %
Psychiatrist	2 %
Occupational therapist	5 %
Counsellor	10 %
Youth worker	21 %
Management	10 %
Other (Aboriginal health, community health, youth project officer)	10 %

*note totals do not add to 100 % as some workers identified with more than one profession

### Procedure

#### Focus groups

Focus groups were chosen as the primary data collection method because they enabled group-level discussion, to unpack and debate personal and professional beliefs and understandings of technology in their work. The focus groups lasted between 1.5 and 2 h in length and were audio recorded. Three of the four focus groups were composed of staff from pre-existing mental health teams working with youth in the region; therefore participants were known to and had working relationships with each other. Focus groups were arranged by emailing the team leader of the service, with composition dependent on staff availability and willingness to participate, and aligning with times designated for team meetings or professional development. Focus groups 1–3 were carried out at the participating service. This approach allowed analysis of both individual and service-level experiences. In order to gain a wide variety of youth sector perspectives, staff in other youth-related services (e.g. education, local government, psychosocial support) were also recruited and participated in one focus group which was carried out at workplace of one of the participants. A preamble at the outset of the focus groups outlined that by technology we were referring to mobile and web-based tools which are usable by the clinician in collaboration with the consumer. Examples of questions included: *How comfortable do you feel using technology in your professional practice? (both philosophically and practically); How could your current comfort level with technology be improved?; In which ways are you currently using technology in your professional practice?; and What are the barriers to your use of technology in your professional practice?*

#### Semi-structured interviews

Participants were approached via email regarding their participation in the interviews. The semi-structured interviews lasted between 1 and 1.5 h in length and were audio recorded. The interviews were carried out with youth mental health clinicians working in three different South Australian rural regions (*n* = 3) and managerial/support staff working in one of the mental health services represented in the initial focus groups (*n* = 5). The interviews sought to explore themes and issues identified in the focus groups in greater detail. The data from the focus groups was then triangulated with data extracted from semi-structured interviews. The interviews were carried out until data saturation was reached and were predominantly at participants’ workplaces (except two; one of which was conducted at a café and the other at the researchers’ workplace).

All focus groups and interviews were conducted by the first author.

#### Member checking workshop

To increase validity of the findings, member checking was also carried out via a participatory workshop [44]. Individuals who participated in the original focus groups/interviews were invited to attend the workshop and 14 of the original 48 study participants chose to. During the workshop, draft researcher-determined themes and sub-themes were provided as a starting point and, in groups of 4–5, participants produced their own thematic maps. This involved adding, deleting and repositioning themes where participants deemed it appropriate.

### Sampling frame

In order to gain an in-depth insight from a diversity of workers’ views, participants were recruited using a maximum variation sampling approach [45]. This approach involved sampling views from a small number of cases that represent the diversity relevant to the role of technology in youth mental health services [45]. Sample size was determined when saturation of ideas was reached, as determined by the research team during data analysis discussions [46].

### Data analysis

Focus groups and interviews were professionally transcribed (and checked for accuracy by the first author). Transcripts were then analysed using inductive thematic analysis [47] using NVivo software [48] to manage the data. The analytic process described by Braun and Clarke [47] was adapted for the current purpose. Initially, this involved: 1. Reading and re-reading of transcripts; 2. Generation of initial codes; 3. Searching for themes; and 4. Reviewing themes and production of a thematic map. To increase the validity of the results, steps 1–4 were independently carried out by first and fifth authors. The resultant thematic maps were then compared for consistency and an overall map was produced. These themes were then member checked at a participatory workshop (as described above). The first author then carried out Step 5. Defining and naming themes – the final themes aimed to represent the various interpretations of the data. The second author then provided a logic check regarding finalisation and parsimony of the themes.

## Results

Six major themes emerged from the data. Direct quotes from participants are used to demonstrate each theme (see Additional file 1 for detailed examples of quotes from participants that demonstrate each of the themes discussed). Together, they represent: an overall picture of the digital world in which young service consumers now live (theme one); the enablers and challenges this is perceived to create for delivery of mental health care and the therapeutic relationship with consumers (theme two); how technology potentially changes and challenges traditional health professional expertise and interactions with consumers, shifting the power base (theme three); how workers then variously respond to and cope with these challenges (theme four); workers’ underlying technology literacy that shapes their response to these challenges (theme five); and, the role of the organisation in addressing these challenges (theme six).

### Young people in a digital age

Perceptions of the preferences, motivations and reality of young people’s lives were central to discussion of the role of technology in youth mental health services. Participants saw technology as a central part of young people’s lives. This was perceived to have implications for the way consumers engage with the clinician and the service and the types of therapeutic conversations that occur between consumer and clinician. There was the general sense that young people are *born into* technology. Some workers suggested that technology could have a positive impact on young people, such as promoting opportunities for connection, belonging and support. Overall, however, there was a strong feeling that reliance on technology could have both direct and indirect effects on youth health and wellbeing via cyberbullying, addiction to technology and increasingly limited face-to-face communication. It created a new and complex world in which to provide mental health care, with advantages and disadvantages and an unclear sense of how to control for these within their role. Participants’ comments highlighted generational differences that they perceive can exist between a clinician and consumer, with clinical best practice involving the ability to appreciate and work within these differences. Participants also spoke about the fine balance between being relatable as a clinician and appearing too eager to appear on a young person’s level.

### Personal connection

Discussions around the role of technology in youth mental health services highlighted the centrality of human interaction and connection in the provision of effective mental health services. Technology was seen to simultaneously enhance and restrict this central component of service provision, with a complex series of implications and consequences. There was general consensus among participants that technology-based interaction filters communication in a way that does not assist clinical practice because the latter relies heavily on non-verbal consumer cues and the ability to develop a strong therapeutic rapport via personal connection. For this reason, participants viewed technology as an adjunct only to face-to-face practice. This was also linked to the notion that therapy offers an opportunity for young people to extract themselves from technology and to develop skills required for in-person communication, and active promotion of technology in clinical work may hinder this.

Despite these reservations, participants perceived value in technology for its ability to promote connection and enhance the vital consumer-clinician relationship in previously impossible ways. Short Message Service (SMS) and email (and rarely Facebook chat/messaging rarely) was reported as currently used to connect with consumers outside of sessions; particularly around appointment organisation or reminders. These practices were linked to consumer preferences and awareness of the limited financial freedom most young people face in making phone calls. In the rural context, this technological value took on particular importance. Despite outreach provided by each of the services, consumers living in more remote locations (i.e. outside of larger rural centres) were seen as particularly vulnerable to experiencing extended wait times between appointments, due to both the regularity of outreach visits and consumers’ ability to travel to the clinician. Even in larger rural towns, public transport options were restricted for many consumers. Moreover, participants expressed a desire to engage more readily with consumers’ families as this was a seen a current area of weakness in service delivery. Participants acknowledged that many young consumers deal with intergenerational familial problems and, despite often complex relationships, family was perceived to occupy a powerful support role in young peoples’ lives. Current/emerging technologies were suggested as a means by which increased information sharing and/or shared use of therapeutic technologies outside of session could occur. The same was also true for schools, counsellors, peers/friends and other services that may be a part of the young person’s help seeking experience.

### Power and vulnerability

When analysing the role of technology in face-to-face youth mental health services, power and vulnerability underpinned a number of perspectives across the data. The concepts were referenced directly in the suggestion that greater use of technology with consumers questions the historical location of power between clinician as expert and consumer as recipient of care. The digital age was seen to be disrupting more traditional power structures, opening up new vulnerabilities in how people relate to each other, and altering the assumed capacities for each party to exercise control within these environments depending on the extent and form of their engagement with technology. Furthermore, for some youth, it was noted that acknowledgement and demonstration of their digital literacy and skills may provide an opportunity for the young person to occupy a position of power in their family which had not previously existed.

Power and vulnerability were also referenced indirectly in discussions around language and technology. Participants said that their ability to connect and form a strong therapeutic bond with a consumer required them to engage in meaningful conversations around the young person’s experience. Language was seen to be crucial in this process. Awareness of popular social media sites/applications and games such as Snapchat was seen as an important way to promote engagement with young people. Some participants asserted that fluency with current technical language was not necessary; however, a willingness and curiosity to seek clarification and learn from the young person was useful to the relationship. In other instances, participants’ perceived mastery of relevant language as linked to clinicians’ credibility in the eyes of the young person. This tension between perspectives highlights the professional implications and vulnerabilities inherent in greater technology use in youth mental health services. Moreover, in further exploring some clinician concerns around use of technology (i.e. concerns around increased clinical risk and confidentiality/privacy), non-clinicians noted that some of the perceived resistance to technology may have been masking vulnerabilities around exposure of limited skill and confidence in application of technology in clinical work.

Clinicians generally talked about being familiar with technology (i.e. knowing both *how* and *that* it works and is recommended), which spoke to a personal acceptance of the technology and a need for congruence with their professional practice. In contrast, the non-clinical/management staff talked more specifically about skill and confidence when using technology in front of others such consumers and peers (i.e. overcoming fear of the unknown and impression management). These two views were identified as similar but different ways of talking about control – the ability for the clinician to manage the way in which technology is (or is not) integrated into their practice and equally the extent to which they desire to appear in control of their work.

### Professional identity

In a number of different ways, participants explored, questioned and asserted their professional identities when discussing the impact of technology in youth mental health services. The professional identity theme manifested in discussions around when, why and how technology should be used by clinicians and consumers. In-person service provision was foundational to the participants’ role identity. Participants described a definite time and place for technology in their work and indicated that technology has a role both in and outside of session and as a bridge between the two. When appropriately resourced (i.e. having a phone, tablet or laptop and internet connection in-session), some participants reported using technology with consumers to increase engagement and develop rapport. Others reported using technology to access websites such as *beyondblue* [49] to provide psychoeducation to consumers and their families and, to a lesser extent, recommending client use of mobile apps such as Smiling Mind [50] or websites such as MoodGYM [51]. Technology was deemed to have significant potential to engage young people by tracking clinically important neuro-vegetative markers, such as sleep, diet, mood, energy, concentration. Other novel uses of technology included social media genograms and systematically assessing a young person’s technology use - one clinician broadly termed this a “Media Diet*”* and used systematic questioning around online and general media consumption to determine its impact on mental health and wellbeing.

Furthermore, workers described a distinct set of conditions that would facilitate their use of technology in professional practice. This included personal familiarity with the technology in question, accessibility to resources in-session such as hardware and an internet connection, and a desire for a clear evidence base and recommendation by reputable individuals and/or services. However, keeping up with the myriad of options available and the break-neck pace of technological innovation was seen as a barrier to uptake in clinical practice. Ultimately, workers reported that they needed more time to feel comfortable and prepared in use of technology-based applications and systems as an adjunct to their clinical practice. This included the need for: 1. Reliable internet access - which can be limited in terms of connection and cost for some rural youth consumers; and 2. Consumer interest and willingness to engage with technology as part of their engagement with the service.

Professional identity, expressed through face-to-face service provision, was also tied to discussions of risk. In particular, asynchronous technology-mediated communication was associated with risks in inaccurately assessing a consumer’s mental state, and an increased possibility of miscommunication or misinterpretation. Clinicians were very clear on the implications of their professional and legal responsibility to accurately assess consumers’ level of risk. This was linked to the importance of non-verbal cues derived from face-to-face engagement with consumers. Dealing with risk was identified as a major focus of training for mental health clinicians; introducing technology, as a way of engaging with consumers, was viewed as potentially increasing the risks a clinician must contend with. Furthermore, maintaining confidentiality of consumer data was seen as central to the work of youth mental health services. For example, the use of technology as an adjunct to clinical practice raised concerns around how and where data would be stored and the confidentiality of clinicians’ identity (e.g. giving out email addresses or communication over social media). This limitation to technology was perceived as a particular concern in the rural setting in which personal and professional boundaries can and do blur. Concerns were also raised around the utility of some technologies and their applicability in working with some clinical presentations (e.g. concerns around increasing access to clinicians for consumers with a borderline personality disorder diagnosis or the appropriateness of technology when working with consumers with a diagnosis of schizophrenia). There was also a feeling that clinician and service use of technology with consumers might implicitly encourage overuse or reliance on technology.

### Individual factors

Individual worker factors such as age, personal attitudes/beliefs, preferences and experiences heavily influenced their perceptions of the role of technology in youth mental health services and society more broadly. For most participants, prior experience with and use of technology in their personal lives translated to an increased willingness to experiment with or use technology in their clinical practice. There were, however, examples of dissenting cases both where personal use did not translate into professional use and limited personal use did not prohibit professional use. Moreover, older age was linked to unfamiliarity and inexperience with technology and a difference between the way in which older generations communicate and view/interact/use digital and ICT-based technologies compared with today’s youth. Participants expressed a general belief that ICT and digital technologies are an increasingly important part of modern society – with the ability to make daily activities/routines faster and easier. Personal preferences around face-to-face service provision, however, seemed to be associated with a belief that technology limits the quantity and quality of face-to-face connection. This belief was linked to observed personal consequences such as reduced social connection, engagement and resilience. These consequences were often linked to the internet and social media and particularly their negative aspects such as obsessive use, cyberbullying and general perpetuation of negative and anti-social behaviours. A minority of participants rejected these views and asserted that technology only reflects the wider social experience. As discussed earlier, technology was generally seen as a way of interacting with young people on their terms.

### Organisational legitimacy

Participants discussed a number of organisational factors that, taken together, suggested a need for legitimacy in any technology-related change to youth mental health services. These factors included appropriate organisational priorities, policy, systems and structures to support use of clinically appropriate and useful technologies that are integrated into current practice. Use of technology in practice was not seen as the sole responsibility of individual workers. Technology adoption was linked to organisational budgets, built around organisational priorities and strategic direction, which are largely determined by outcomes and resourcing the organisation values and promotes. It was clear that clinical outcomes and cost savings were important in encouraging large-scale investment in the required resources. The term “culture change” was used in reference to use of technology with consumers. When discussing the role of technology in services some participants pointed to prior negative experiences with technology such as a lack of streamlining between organisational databases. Non-clinical/managerial staff discussed the need to understand current work practice(s) in order to build supportive structures and business systems.

Organisational legitimacy was also reflected in participants’ expressed need for clear and detailed organisational policies and procedures to govern and drive use of technology in practice. However, non-clinical/managerial staff generally felt that excessive policy-making might hinder staff innovation and promote prescriptive work practices. These dichotomous perspectives speak to assumptions around risk – both in terms of what is seen as risky and the level of personal versus organisational responsibility desired in promotion of innovative practices. In some instances, current policy prevented participants from downloading apps and various programs onto organisational assets which conflicted with a desire by management for innovation.

Use of technology in clinical practice was also linked to use of SMS, email, social media and applications for tracking clinically relevant indicators such as mood and sleep. While the benefits of technology were seen in the ability to more closely track consumers’ progress and level of risk, it was also associated with concerns around an implied level of clinician responsivity and possible increases to workload. These concerns included a perceived lack of processing around information communicated via these modes of communication and unclear guidelines around when and how clinicians should respond to potentially risky information, particularly outside of work hours. Some participants discussed guidelines they negotiated with consumers to establish clear expectations of when and how the clinician would respond. Overall, clinicians reported experiencing only minor abuses of the system.

## Discussion

The results of the current study demonstrate the multitude of factors that are at play for mental health workers when considering whether, and how, to use technology to engage youth consumers. While some are internal factors, others are not because they concern organisational and discipline-wide issues. Consistent with other studies in the field [11, 13, 19, 22, 23, 25, 26], the current results indicate that, while some community-based mental health professionals are using technology to engage consumers, it is not currently standard practice. In general, the workforce positions technology as an adjunct tool to complement standard practice, with face-to-face modes of working occupying a central role in service delivery. The low rates of technology use found in the current sample of community-based youth mental health professionals fits with findings from studies with CBT therapists, youth workers and private rural healthcare practitioners [11].

The current results demonstrated overall resistance to technology-related changes based on a clear preference for development of a *personal connection* through face-to-face engagement with consumers; clinicians perceived their skillset as tied to this way of working. These results, along with the results of prior studies, resonate with findings from the earliest sociotechnical studies. The idea that every technology participates in, and contributes to the definition of a sociotechnical system has highlighted that no technical change in people’s workplace could have only instrumental consequences (such as, for instance, increased efficiency), but also has effects on things like workers’ autonomy, morale and professional identities [52–54]. These early sociotechnical workplace studies, along with the current results, continue to underline the importance of autonomous, adaptable, complex and meaning-driven work for the creation of successful technologically-mediated workplaces. More recent sociotechnical research highlights the importance of degree of fit between technical design and level of local control and flexibility afforded the individuals in working with the system [55].

### Implications for work practice

Individual beliefs, preferences, experiences and work practices were shown to impact on use of technology in community-based mental health practice. Similar to prior research [26], most participants viewed integration of technology as *extra work* for which they were under-resourced. This took the form of: 1. Physical resourcing - internet enabled devices and internet connections required to bring the technology where it is needed (i.e. for use with consumers) are generally unavailable; 2. Time - clinicians have limited time to remain abreast of the technologies available, to familiarise themselves with individual technologies and to be confident regarding their evidence base; and 3. Skills and training - the majority of clinicians felt undertrained and skilled in the use of technology to engage consumers. With this in mind, recent investment in workforce training and support, along with redefining clinical practice models, to facilitate technology integration into mental health work is most welcome [11].

With respect to the proposed technological change to youth mental health services, the *power and vulnerability* theme highlights the implications of, and on, the socio-political landscape. Mental health clinicians have been socialised and trained in an ‘expert’ role which, in turn, positions consumers as (largely passive) recipients. Whilst there is a definite policy trajectory toward more empowered consumers [56], the day-to-day practice of mental health service provision sits squarely in an expert-recipient model. Technology-based engagement with consumers has the potential to actively shift the power away from clinicians toward consumers. The vulnerabilities and uncertainty inherent in this shift are best made visible in the references to language present in the results of the current study. References to lack of clinician fluency with terminologies associated with use of digital applications and social media popular with youth, speak to a gap in knowledge and skills. The mental health workforce would need to work through this gap in order to enact the required practice change. Engaging with consumers via technology creates tensions between traditional ways of working and a growing appreciation for more consumer-centred approaches. These results have been echoed elsewhere with suggestions that “intrinsic judgements of acceptability and the expectations accompanying their socially defined role” impact on a clinician’s decision to use and/or refer clients to technology-based tools and supports [26].

Similarly, participant concerns around confidentiality and legal and professional implications of technology are based in shared disciplinary perceptions of risk and the consequences of challenging longstanding ways of communicating and engaging with consumers in healthcare that are articulated in discipline-specific codes of practice [57]. Health service provision is inextricably linked to risk [27–29] which is understandable considering the duty of care shouldered by professionals and organisations under legislation. The results of this study, particularly within the *professional identity* theme, are a manifestation of this orientation to risk. The results suggest that use of technology with consumers was linked to a perceived increased risk of: 1. Inaccurately assessing mental status; 2. Increased workload as a result of implications of increased responsivity; 3. Being exposed with limited technological literacy; 4. Professional consequences linked to technology enhanced work practices; and 5. (In)ability to maintain clinician and consumer confidentiality. More broadly, the clear majority of participants felt that technology-mediated communication filters the human experience and that the rise of social media, in particular, has adversely impacted societal engagement and communication, a phenomenon they feared perpetuating through use their work. It is in this context that the preference for face-to-face engagement with consumers should be understood.

ICT implementation evaluation studies suggest high failure rates, coupled with incomplete adoption of the technology [58]. As in the current context, this situation can be exacerbated where technology is not integral to work functions and users have a choice about how and when they use it. It has been suggested that “by achieving the right balance and designing processes and policies that recognise the interdependency between the social and technical subsystems of an organisation, the performance of an organisation can be optimised” [59]. With respect to the current study, the *organisational legitimacy* theme clearly highlights the need for organisational leadership around how: 1. Technology will be introduced (and why); 2. The impact on work roles will be negotiated; and 3. The impact on work roles will be accounted for. Moreover, the conflicting perspectives between workers and management over the role of policy in promoting uptake in practice, present in this study and others, highlight tensions around the level of organisational permission and support necessary for innovation to flourish [19]. While some clinicians, generally those who value and use technology in their private lives, are willing to assume personal responsibility for negotiating safe and appropriate engagement with consumers via technology, others are not. These sentiments have also been raised in the telehealth literature where the notion of legitimacy was important factor in successful implementation and uptake. As such, providing healthcare services via videoconferencing facilities needed to be seen as safe, normal and part of routine practice; this way of working also needed to be supported by established protocols and standards [20]. Recent research suggests that, despite a significant body of literature around efficacy of technologies such as iCBT’s, routine uptake of these or many other technologies is not supported by appropriate research around appropriate financing, governance and implementation models [10]. The reticence toward technology evident in the current study’s results appears, in part, to be linked to this lack of sector-wide leadership.

### Implications for design and implementation of technology

As the results of this study and others suggest [13, 19], technology-based change to work roles and practice needs to be seen as legitimate. Designing with those intending to use and those required to resource and promote the output can help to achieve this. Participatory Design [60] in mental health has been explicitly recommended [61] and the results of this study and others [13, 19] provide additional support for this recommendation. Design that is embedded in the workplace is crucial because, as the results of this study indicate, widespread adoption of technology must be championed from the highest levels of an organisation – those responsible for strategic direction and budget allocation. These ideas around legitimacy are exemplified in the current study results which highlight the need to create and change business systems to support change in practice. Beyond participatory design, approaching implementation in a transitional way via localised piloting/soft rollouts of technology can help to work through the complex reality of technology-related change. It can do this by allowing adoptees to make sense of, and gain ownership over, the technology-related change and to suggest necessary changes/improvements [58, 62–64]. The notion of designing for minimum specification is important for *legitimising* technology in workplaces.

This process can be encouraged by complementing *organisational inquiry* with *problem closure* in design [64]. This complementary process seeks to balance designing a solution to ‘fix’ a pre-defined problem with seeking to understand, via consultation, what the problem is in the first place [64]. When problem definition is predefined by designers and selected stakeholders in *problem closure* only projects (which are common), these projects are susceptible to failing to meaningfully recognise the central role of the sociotechnical system in its eventual success. Organisational inquiry via inclusive and consultative goal setting is crucial. Workplace technology implementation projects often struggle due to different, and often conflicting, goals of the various stakeholders and intended users in any given project, even when they seem aligned from the outset [65, 66]. Therefore a well-defined process that considers and balances all stakeholders’ needs, desires and preferences when setting projects goals is suggested (Gasson, 2003). These goals need to be regularly revisited throughout the life of the project to ensure fidelity or to gain a consensus for change [62, 64, 65].

### Limitations

The current study has a number of limitations. Initially, young people’s perspectives were not represented in this study. However, the data used in this research represents only one arm of a larger ongoing study in which workshops and semi-structured interviews were also undertaken with rural young people. The decision to conduct focus groups within existing mental health service teams was made in order to approximate naturally occurring discussions, with the benefit of participants being able to connect with one another’s stories and experiences and often question each other in ways not possible if participants were unknown to each other [67]. Conducting focus groups in this way, however, introduces different power dynamics, as hierarchies exist between staff members. These power differences were offset by enabling participants to exclude questions specifically asking them about their workplace, employee relations and job conditions/satisfaction over and above being asked for general comments about working rurally, so participants were less likely to be censored in their responses. The fact that the dedicated mental health services in the region work largely independently but are keenly aware of one another meant that, even if focus groups were carried out with participants from different services, it is unlikely they would have been unknown to each other given the rural context of the research. With this in mind, this possible limitation could also be considered a major strength of the study as it allowed a variety of perspectives to be sought and debated, which resulted in the rich data set yielded. Despite perspectives being sought from different rural regions, most of the data was collected in one region. Whilst this design allowed for in-depth data collection and analysis, the results should be understood in this context. Similarly, the data collected from executive-level management personnel was the result of sampling one mental health service working with youth. Finally, in the interests of curtailing the study to a manageable population, the youth mental health service workforce did not include general practitioners or those working in private practice such as psychologists and psychotherapists.

## Conclusion

The adoption of technology-based consumer engagement tools by youth mental health clinicians and services involves a major practice change, one that is not currently supported or prioritised by individuals, organisations or the mental health sector more broadly. Nor is it currently likely given the radical cultural transformation that is required to achieve widespread adoption of technology. The culture required to support such a practice change requires a historical appreciation of the challenges of technology adoption that accounts for individual, organisational and discipline-wide perspectives. Technology is revolutionising mental health care. The question with which policy makers, organisations, clinicians and academics are now faced is both how and whether we will work together to make the most of this.

## Additional file

Additional file 1: Supporting quotes. Supporting quotes for six themes outlined in the results. (DOCX 19 kb)

## Abbreviations

ICT: Information communication technology
SMS: Short message service

## References

[1] McGorry PD, Purcell R, Goldstone S, Amminger GP (2011). Age of onset and timing of treatment for mental and substance use disorders: implications for preventive intervention strategies and models of care. Curr Opin Psychiatry.

[2] Ivancic L, Perrens B, Fildes J, Perry Y, Christensen H (2014). Youth mental health report.

[3] Clement S, Schauman O, Graham T, Maggioni F, Evans-Lacko S, Bezborodovs N, Morgan C, Rüsch N, Brown J, Thornicroft G (2015). What is the impact of mental health-related stigma on help-seeking? A systematic review of quantitative and qualitative studies. Psychol Med.

[4] Rickwood DJ, Deane FP, Wilson CJ (2007). When and how do young people seek professional help for mental health problems?. Med J Aust.

[5] Australian Government. e-Mental Health Strategy. 2014. http://www.mindhealthconnect.org.au/emental-health-strategy. Accessed 3 Sept 2015.

[6] Burns J, Birrell E (2014). Enhancing early engagement with mental health services by young people. Psychol Res Behav Manag.

[7] Christensen H, Hickie IB (2010). Using e-health applications to deliver new mental health services. Med J Aust.

[8] Eysenbach G (2001). What is e-health?. J Med Internet Res.

[9] Griffiths KM, Christensen H (2007). Internet‐based mental health programs: A powerful tool in the rural medical kit. Aust J Rural Health.

[10] Meurk C, Leung J, Hall W, Head BW, Whiteford H (2016). Establishing and governing e-Mental Health Care in Australia: a systematic review of challenges and a call for policy-focussed research. J Med Internet Res.

[11] Reynolds J, Griffiths KM, Cunningham JA, Bennett K, Bennett A (2015). Clinical practice models for the use of e-mental health resources in primary health care by health professionals and peer workers: a conceptual framework. JMIR Ment Health.

[12] Hilty DM, Ferrer DC, Parish MB, Johnston B, Callahan EJ, Yellowlees PM (2013). The effectiveness of telemental health: a 2013 review. Telemed J E Health.

[13] Montague AE, Varcin KJ, Simmons MB, Parker AG. Putting technology into youth mental health practice. SAGE Open. 2015;5(2):2158244015581019.

[14] Australian Bureau of Statistics. Household use of information technology, Australia 2014–15. 2016. http://www.abs.gov.au/ausstats/abs@.nsf/mf/8146.0 Accessed 3 Sept 2015.

[15] Al-Qirim NAY (2003). Teledermatology: the case of adoption and diffusion of telemedicine health Waikato in New Zealand. Telemed J E Health.

[16] Koivunen M, Hätönen H, Välimäki M (2008). Barriers and facilitators influencing the implementation of an interactive Internet-portal application for patient education in psychiatric hospitals. Patient Educ Couns.

[17] Sadasivam RS, Delaughter K, Crenshaw K, Sobko HJ, Williams JH, Coley HL, Ray MN, Ford DE, Allison JJ, Houston TK (2011). Development of an interactive, web-delivered system to increase provider–patient engagement in smoking cessation. J Med Internet Res.

[18] Whitten P, Kuwahara E (2004). A multi-phase telepsychiatry programme in Michigan: organizational factors affecting utilization and user perceptions. J Telemed Telecare.

[19] Blanchard M, Herrman H, Frere M, Burns J (2012). Attitudes informing the use of technologies by the youth health workforce to improve young people’s wellbeing: Understanding the nature of the “digital disconnect”. YSA.

[20] Wade VA, Eliott JA, Hiller JE (2014). Clinician acceptance is the key factor for sustainable telehealth services. Qual Health Res.

[21] Wade V, Eliott J (2012). The role of the champion in telehealth service development: a qualitative analysis. J Telemed Telecare.

[22] McMinn MR, Bearse J, Heyne LK, Smithberger A, Erb AL (2011). Technology and independent practice: Survey findings and implications. Prof Psychol Res Pract.

[23] Simms DC, Gibson K, O'Donnell S (2011). To use or not to use: Clinicians’ perceptions of telemental health. Can Psychol.

[24] Boydell KM, Greenberg N, Volpe T (2004). Designing a framework for the evaluation of paediatric telepsychiatry: a participatory approach. J Telemed Telecare.

[25] Cloutier P, Cappelli M, Glennie JE, Keresztes C (2008). Mental health services for children and youth: a survey of physicians’ knowledge, attitudes and use of telehealth services. J Telemed Telecare.

[26] Sinclair C, Holloway K, Riley G, Auret K (2013). Online mental health resources in rural Australia: clinician perceptions of acceptability. J Med Internet Res.

[27] Ryan C, Nielssen O, Paton M, Large M (2010). Clinical decisions in psychiatry should not be based on risk assessment. Australas Psychiatry.

[28] Sawyer A-M (2005). From therapy to administration: Deinstitutionalisation and the ascendancy of psychiatric ‘risk thinking’. Health Sociol Rev.

[29] Rose N (1998). Governing risky individuals: The role of psychiatry in new regimes of control. Psychiatry Psychol Law.

[30] Furber GV, Crago AE, Meehan K, Sheppard TD, Hooper K, Abbot DT, Allison S, Skene C (2011). How adolescents use SMS (short message service) to micro-coordinate contact with youth mental health outreach services. J Adolesc Health.

[31] Gardner W, Klima J, Chisolm D, Feehan H, Bridge J, Campo J, Cunningham N, Kelleher K (2010). Screening, triage, and referral of patients who report suicidal thought during a primary care visit. Pediatrics.

[32] Mailey EL, Wójcicki TR, Motl RW, Hu L, Strauser DR, Collins KD, McAuley E (2010). Internet-delivered physical activity intervention for college students with mental health disorders: a randomized pilot trial. Psychol Health Med.

[33] Reid SC, Kauer SD, Hearps SJC, Crooke AHD, Khor AS, Sanci LA, Patton GC (2013). A mobile phone application for the assessment and management of youth mental health problems in primary care: health service outcomes from a randomised controlled trial of mobiletype. BMC Fam Pract.

[34] Sethi S, Campbell AJ, Ellis LA (2010). The use of computerized self-help packages to treat adolescent depression and anxiety. J Technol Hum Serv.

[35] Searl MM, Borgi L, Chemali Z (2010). It is time to talk about people: a human-centered healthcare system. Health Res Policy Syst.

[36] Lord J, Dufort F (1996). Power and oppression in mental health. Can J Commun Ment Health.

[37] Matthews B, Heinemann T (2009). Technology use and patient participation in audiological consultations. AMJ.

[38] Mullaney T, Pettersson H, Nyholm T, Stolterman E (2012). Thinking beyond the cure: A case for human-centered design in cancer care. Int J Des.

[39] Nelson G, Kloos B, Ornelas J. Community psychology and community mental health: Towards transformative change. New York: Oxford University Press; 2014.

[40] Nelson G, Lord J, Ochocka J. Empowerment and mental health in community: Narratives of psychiatric consumer/survivors. J Community Appl Soc Psychol. 2001;11(2):125–142.

[41] Garland AF, Plemmons D, Koontz L (2006). Research–practice partnership in mental health: Lessons from participants. Adm Policy Ment Health.

[42] Government of South Australia SA Health. South Australian Youth Mental Health System of Care. 2012. https://www.sahealth.sa.gov.au/wps/wcm/connect/c9c5f480437f731382659f0a3dc25030/Youth%2BMental%2BHealth%2BSystem%2Bof%2BCare%2BFramework%2BMay%2B2012.pdf?MOD=AJPERES&CACHEID=c9c5f480437f731382659f0a3dc25030. Accessed 3 Sept 2015.

[43] Australian Bureau of Statistics. Main Features - South Australia ACA, 2015 Main Features - South Australia, Australia 2011–12. 2015. http://www.abs.gov.au/ausstats/abs@.nsf/Products/3218.0~2011-12~Main+Features~Main+Features?OpenDocument. Accessed 3 Sept 2015.

[44] Creswell JW, Miller DL (2000). Determining validity in qualitative inquiry. Theor Pract.

[45] Grbich C. Qualitative research in health: an introduction. London: Sage; 1998.

[46] Rennie DL, Phillips JR, Quartaro GK (1988). Grounded theory: A promising approach to conceptualization in psychology?. Can Psychol.

[47] Braun V, Clarke V (2006). Using thematic analysis in psychology. Qual Res Psychol.

[48] QSR International Pty Ltd (2012). NVivo qualitative data analysis software (version 10).

[49] beyondblue. https://www.beyondblue.org.au/. Accessed 3 Sept 2015.

[50] Smiling Mind. http://smilingmind.com.au/. Accessed 3 Sept 2015.

[51] MoodGYM Training Program. https://moodgym.anu.edu.au/welcome. Accessed 3 Sept 2015.

[52] Trist E, Bamforth K (1951). Some social and psychological consequences of the Longwall method. Hum Relat.

[53] Trist EL. Organizational choice: capabilities of groups at the coal face under changing technologies: the loss, re-discovery & transformation of a work tradition. Tavistock Publications; 1963.

[54] Rice AK. Productivity and social organization: The Ahmedebad experiment. London: Tavistock; 1963.

[55] Eason K, Waterson P (2013). The implications of e-health system delivery strategies for integrated healthcare: lessons from England. Int J Med Inform.

[56] Australian Government Department of Health. A National framework for recovery-oriented mental health services: guide for practitioners and providers. 2013. http://www.health.gov.au/internet/main/publishing.nsf/Content/mental-pubs-n-recovgde. Accessed 3 Sept 2015.

[57] Australian Government Department of Health. National practice standards for the mental health workforce 2013. 2013. http://www.health.gov.au/internet/main/publishing.nsf/Content/mental-pubs-n-wkstd13. Accessed 3 Sept 2015.

[58] Eason K (2008). Sociotechnical systems theory in the 21st Century: another half-filled glass.

[59] Westbrook JI, Braithwaite J, Georgiou A, Ampt A, Creswick N, Coiera E, Iedema R (2007). Multimethod evaluation of information and communication technologies in health in the context of wicked problems and sociotechnical theory. J Am Med Inform Assoc.

[60] Ehn P (1993). Scandinavian design: On participation and skill. Participatory design: Principles and practices.

[61] Hagen P, Collin P, Metcalf A, Nicholas M, Rahilly K, Swainston N (2012). Participatory Design of evidence-based online youth mental health promotion, intervention and treatment.

[62] Bar-Lev S, Harrison MI (2006). Negotiating time scripts during implementation of an electronic medical record. Health Care Manage Rev.

[63] Venkatesh V, Bala H (2008). Technology acceptance model 3 and a research agenda on interventions. Decis Sci.

[64] Gasson S (2003). Human-centered vs. user-centered approaches to information system design. JITTA.

[65] Orlikowski WJ (2000). Using technology and constituting structures: A practice lens for studying technology in organizations. Organ Sci.

[66] Cornford T, Klecun-Dabrowska E. Telehealth technology: consequences for structure through use. Stud Health Technol Inform. 2001;84(Pt 2):1140–1144. 11604907

[67] Kitzinger J (1994). The methodology of focus groups: the importance of interaction between research participants. Sociol Health Illn.

