# Supplementary material for: The promise and the reality: a mental health workforce perspective on technology-enhanced youth mental health service delivery
Source: BMC Health Serv Res. 2016 Oct 10;16:562. doi: 10.1186/s12913-016-1790-y (PMC5057226; doi:10.1186/s12913-016-1790-y)
Supplement: Additional file 1: — Supporting quotes. Supporting quotes for six themes outlined in the results. (DOCX 19 kb) [file 12913_2016_1790_MOESM1_ESM.docx]

| Illustrative quotes for the major themes |
| --- |
| **Young People In A Digital Age**  To match what is, what the next generation is going to do and what, what sort of my generation did ages ago worked for us, but it may not work for the rest and we might have to adjust it dramatically. (Participant 26)  I think that having that understanding that technology is a part of their life, it is a massive part of their life. (Participant 16)  for many of them they haven't known life without it and I think it's only going to grow and increase um and so I think probably, organisations at some point, need to balance the risk with relevancy and them as the centre. (Participant 38)  Technology can be a source of great stress for them um you know with cyber bullying and all that sort of thing so um and it can be a great source um of strength for them. It can be a real um source of social connection um for them um which all impacts the person's wellbeing. (Participant 31)  You know, we’re not natives. We’re definitely immigrants to this world. (Participant 48) |
| **Personal Connection**  You need to be able to respond to somebody so they feel like there is a human being at the other end of – even if it's a little text or something but there's somebody there that cares about me that I can reach out and connect with even if it's just through a few words. (Participant 39)  If you had an interactive online tool that allows you to stay in touch with your client, particularly if they live out country …….. and you know you have a client there who is you know quite wobbly or has risk issues — so that you have an online chat function with a client. (Participant 34)  It would be really ideal for a parent to be involved in the level that they can help the young person with a thought diary or, or you know sit with the young person and do a meditation exercise or you know like but to have them involved on that online platform in some way would help strengthen the support —you know that already existing family and friends support network of a young person so that they use that support network a bit better with that technology. (Participant 35)  I think one of the difficulties with existence in the modern world is that ah it may actually be hiding a connection - that everyone's connected to everyone else but in the most superficial of ways. And what therapy is, it's almost a step backwards in terms of communication. It allows you to have in-depth, meaningful conversations about what's most important in life or what's most important in relationships or what's most important with children………I feel like that can only really be done in the context of a meaningful, intimate, face-to-face conversation and to try and do that via media, I think is a very risky thing. (Participant 9)  I mean the non-verbals are so vital in our system. (Participant 14)  I was just going to say, technology fundamentally filters the human experience and also humans filter themselves by using technology. (Participant 2)  I feel like it's a real evolution over the past decade that we’re all connected ……but relationally, um it's kind of falling apart. So people are kind of more aware of each other's lives than ever but at the same time, not at all. And so I think yeah, not wanting to conclude on a negative note but that's just something we have to be so conscious of that we think young people are connected or we think as individuals are connected but having 500 and something friends on any sort of social media doesn't actually mean you have any sort of relationship or connectedness to those people. (Participant 26) |
| **Power and Vulnerability**  I'm really conscious at times that I can sound like I don't know what I'm talking about but I use the wrong language so, so like I notice my clients now talk about in-boxing each other and it took me a while to pick up that that's a private message on Facebook and they use in-box as a verb…………….I have a friend who says that she tags things on Facebook when she actually means she posts them on Facebook and I kind of cringe every time she says it, wanting to correct her and I, and I wonder if our clients do that with us, if they're cringing. (Participant 12)  How do you shift power which is, at the moment, predominantly owned by service providers, into a middle space of a collaborative approach, rather than an external recipient set up. (Participant 46)  Like any new technology when you bring that in, people are worried about not, how they’re seen by their peers, whether they be good enough at it or whatever um and then a lot of the excuses you get coming up – that’s when you get the secondary barriers where people will then put up the stuff around confidentiality, around not enough training, around, “It takes me longer to do it,” - (Participant 45) |
| **Professional Identity**  *Ways technology is incorporated into clinical work*  It would be great to have apps and websites where you can start therapeutic work in session. I'm thinking for example, CBT thought diaries, you could, young people could start them online with you in a session and then they could do that work between sessions and you'd have access to that. So you know it removes barriers like people forgetting to bring things to session like if you've got paper worksheets (Participant 35)  I think it can be useful to titrate the social exposure so whilst there can be a way to avoid, it can also be a way – like I've got one client who's very socially anxious sort of to the point of just freezing when you would ask her a direct question so it was useful to take a back step and try and communicate via email. (Participant 11)  Like if we were doing some goal setting, to kind of ignite some passion I might "well let's jump on the internet, let's research what you want to do" (Participant 29)  Smiling Mind I use myself and so therefore I'm quite happy to that share that with clients. (Participant 35)  there’s lots of things that you can access through the internet like …… Beyond Blue, there’s….. Mind Gym, there’s lots that I, I do all the time. (Participant 24)  I've started to include information ah about social networking into genograms with young people. So getting information about who's on there – who's in their family but also who's in their social network, who is on Facebook and within their family, who are they friends with? So often that produces very useful information in terms of quality of relationships, whether a mother has blocked the child, which has happened……. Or vice versa yeah. So sometimes the technology although not directly being used, can be a measure of other things we're interested in, in therapy. (Participant 2)  I send an appointment reminder in the morning um for their appointment and um they'll either reply "yes, I'll be there" "no, I won't" (Participant 40)  *Conditions of use*  Um one of the ah concerns is around whether they're evidence-based and um whether they're you know, therapeutic and all of those sorts of things. (Participant 43)  I think it's wonderful if in the future things go down so that there's greater access to support through technology but just not forgetting those regional young people who don't have access to it and not basing everything around that when there's some that just can't access it. (Participant 38)  I feel like I only recommend if I know the site really well and I know that page on that site really well (Participant 12)  Your day's filled up with seeing clients, doing case notes, making phone calls um and you know you definitely have enough work for the whole day. ………….. that's the barrier for me is kind of finding that time to sit down and go through it. I'd want to use it myself if I could, if it was an app that I could use. (Participant 35)  *Confidentiality*  Make sure that there was…..you know, there was secure ways of, of if you were emailing clients so that we are protecting their confidentiality. (Participant 16)  Actually sometimes have issues around confidentiality too about giving other people your email address. Um so I know with the, the youth they can have an app for everything, you know, like what they eat [laughs] you know, how many calories certain foods…. I don’t see if, if they can resource it themselves that there’s an issue, you know, that’s when yeah I’m, I’m sort of a bit more looking into the confidentiality side, what you can give them. (Participant 16)  *Risk*  If people log into Facebook and leave messages and you don't respond, well then that's very risky so they [management] didn't like the idea. (Participant 6)  Does that outcome, what you write down and document and your interactions and actions………..stand up in a Coroner’s court? (Participant 27)  When I scroll back over my texts I've had very intimate and connecting conversations by texts but I have never had that with a client and I don't think I would even attempt to use that same level of intimacy through text um with a client. ……… it’s because it’s more risky, because I can take risks with my closest friends that they will misinterpret what I say and that together we will make sense of that but I can't take those risks with my clients. (Participant 12)  *Perpetuating a problem/promoting over-reliance*  As a parent it’s scary to see your children so reliant on technology. It centres their whole, their whole world, you know (Participant 16)  there are a lot of young people who are, you know, living in a cyber-world or a gaming world um who are actually, you know, really are making themselves extremely unwell and ah who are not engaging with school or friends and not eating well that is just horrendous. And so um sometimes when we think about like um, you know, gamification – gamification of things, it’s always for me, um there’s always for me just that little bit of thought and risk about again reinforcing some of those things that are in fact really unhealthy for young people. (Participant 45) |
| **Personal factors for clinician**  *Personal preference and experiences*  Yeah. I really like using technology. I think also like I am that way inclined myself, I feel one step removed, comes more naturally to me. (Participant 11)  Even to remember, oh yeah maybe you could look up an app on that, oh er it just wouldn't occur to me because I don't do it in my own life (Participant 32)  If I think about myself being not particularly IT literate or IT interested, one thing I find that I use a lot with clients is I use the internet quite a bit with clients when I'm in session with them we might be talking about something or something they've looked up and I'll look it up then and there with them and then it's a useful point for discussion. Um, but I feel like that's a different use of technology, that something that…… deepens the conversation rather than sort of eh shortens the conversation. (Participant 9)  *Attitudes/Beliefs*  It’s a way of life, like you’ve got to incorporate it or you just, you know, get left behind. (Participant 44)  I went to a Fringe [music festival] event, this is a few weeks ago………. I was the only person there not fiddling with my phone. And they weren't there turning it off, they were all fiddling …….. I just feel that's a very sad reflection on how - how intrusive technology has become and how addicted to it we've become. (Participant 9)  This is a business of relationships and it’s a business of narrative, and needing an interpreter to communicate with a person who speaks a different language is, makes it really difficult………. and that interpreter is um, is, is the technology. I’m not saying that’s necessarily a good or a bad thing, but, but I think that’s the discomfort perhaps that we’re feeling about that. (Participant 1)  I don't think that it can work by itself in isolation, I think that you need all different forms of ways of communicating.(Participant 39)  *Age*  The workforce in mental health is average around 50 or thereabouts; it is a group of people who are not as accomplished using media as a younger generation would be. (Participant 46) |
| **Organisational Legitimacy**  *Priorities*  So, any sort of cultural change, it needs to come from the top down. (Participant 44)  So the strategy that we’re thinking about is that we’re trialling it in a, in a semi controlled and reasonable way with the part of our service that makes the most sense – the youth component and that we hope that we can demonstrate something really strongly that gives us a strong business case. (Participant 45)  This whole transforming healthcare imperative that is happening at the moment, there is a recognition that investing in that area may well be worthwhile in terms of improving clinical outcomes and saving costs at the same time. (Participant 46)  *Policy*  Generally speaking, I think policy papers have much more to do with risk management than with enhancing the – sort of, moving forward in terms of opening up new creative spaces. (Participant 46)  Organisational policies can't keep up with the internet and so people will find different ways in which to, to use the benefits of it really (Participant 6)  We don’t want to be overly prescriptive and you can go way too far in terms of um, in terms of prescribing how people do and don’t work around things like technology, there does need to be, there does need to be some framework of support around that. That might be things like policy procedure like a worksite instruction…… guidelines. (Participant 45)  *Professional implications and expectations*  What I've found with a lot of technologies and Facebook and Twitter and all this stuff ……..there could be a risk um if you were receiving that kind of constant feedback information from um that you know where does that leave us around responsibility to respond to what we're seeing. (Participant 30)  Yeah. I really like using technology…... But I always have my Out of Office on and I always make it clear that I can't um respond and at my other job we use text quite a lot but always just make it clear that you can't respond back to this number and um we're only open during these hours. (Participant 11)  *Clinical utility and appropriateness*  So yeah, if you can be smart about it, you know, how’s it actually going to help clinicians in what they have to do, well it will be a winner. (Participant 44)  Talk to teams about the benefit, get teams’ feedback because they’re the experts on how – what’s going to work for them. ……… really involve them in the process, ‘cause if they feel like they can have a part of how it’s going to work they really start to own it. (Participant 44)  Yeah and you can’t always um put a technology in and expect it to just happen itself. We learned that with [e-health initiative] implementation too and so um you can’t just put it in and think it’s going to work. You need a lot of time to create the business systems, to change the business systems to support the process (Participant 45) |
